# Direct Sintering Behavior of Metal Organic Frameworks/Coordination Polymers

**DOI:** 10.1021/acsomega.2c05732

**Published:** 2022-11-18

**Authors:** Izuru Miyazaki, Yumi Masuoka, Akitoshi Suzumura, Shinya Moribe, Mitsutaro Umehara

**Affiliations:** Toyota Central R&D Labs., Inc., Nagakute, Aichi480-1192, Japan

## Abstract

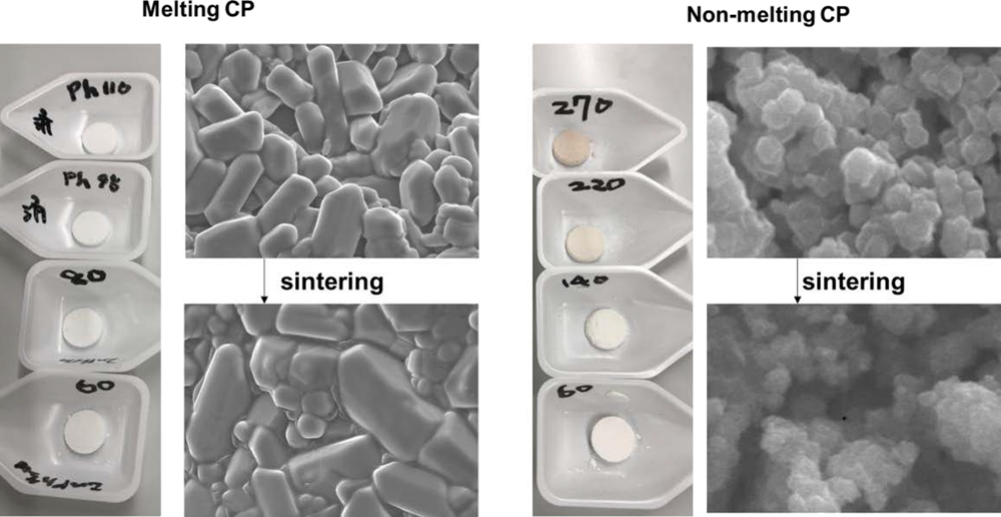

In this study, we investigate the sintering behavior and mechanisms of metal–organic frameworks/coordination polymers (CPs) through physical and microstructural characterization of [Zn(HPO_4_)(H_2_PO_4_)_2_]·2H_2_Im (ZPI; a melting CP, Im = imidazole) and ZIF-8 (a non-melting CP). By performing simple compaction and subsequent sintering, a bulk body of CPs was obtained without losing the macroscopic crystallinity. The sintering behavior was found to be dependent on the temperature, heating rate, and physical properties of the CPs and, in particular, their meltability. During sintering, shrinkage occurred in both the CPs, but the observed shrinkage rate of the ZPI was in the 10–20% range, whereas that of the ZIF-8 was less than 1%. Additionally, the sintering mechanisms of the ZPI and ZIF-8 varied between low and high temperatures, and in the case of ZPI, localized melting between the primary particles was the dominant mechanism on the high-temperature side. However, substantial shrinkage did not correspond to an increase in density; on the contrary, a decrease in the apparent density of ZPI was observed as the sintering temperature was increased. The sintering technique is well established and commercially available; thus, the results obtained in this study can be utilized for optimizing the manufacturing conditions of melting CPs.

## 1 Introduction

Coordination polymers (CPs), especially metal–organic frameworks (MOFs) and porous CPs, have received significant attention in recent years owing to their gas adsorption, catalytic, sensing, and optical properties.^1^ Synthesized CPs are often obtained as fine particles;^2^ however, the powder form is difficult to handle industrially, especially in the case of fluctuations in load, temperature, and pressure.^3^ Therefore, their molding into a shape with easier handling is required. The most common molding method involves the formation of films or sheets using appropriate substrates. Studies on various molding techniques, such as layer-by-layer,^4−7^ in situ synthesis,^8−10^ sputter/atomic layer deposition,^11^ chemical solution deposition,^12,13^ and chemical vapor deposition,^14^ have been conducted. Moreover, several surveys on these studies have been conducted.^15−18^ Furthermore, Küsgens et al. reported the preparation of the CP bulk by extrusion molding of the HKUST-1 powder,^2^ while Hong et al. reported that bulk MIL-101(Cr) can be molded in a similar manner.^19^ Tian et al. reported a more convenient method of mildly drying a solution containing ZIF-8 particles to yield a rigid bulk ZIF-8.^20^ Similar results have been reported for ZIF-8^21^ and ZIF-71.^22^ Bazer-Bachi et al. also prepared bulk ZIF-8, SIM-1, and HKUST-1 by simple powder compression.^23^ Hou et al.^24^ and Duan et al.^25^ have reported more details on the bulk formation of CPs. In recent years, several meltable CPs have been discovered, making the formation of bulk CPs via casting viable.^26^ Some of these can be recrystallized by annealing; thus, CPs with the desired shape can be obtained even in applications where a crystalline product is required.^26^ Research on techniques such as the creation of bubble-free bulk has also been conducted,^27^ and recent studies have exhibited possibilities for the fabrication of new MOF composites from meltable CPs.^28−31^ Horike et al.,^32^ Ma and Horike,^33^ and Bennett and Horike^34^ have provided detailed descriptions of melting CPs.

The direct sintering of crystalline powder is a molding method that is often used in the field of metals and ceramics; however, very few studies have applied it to CPs. In one such study, Widmer et al. recently reported the molding of ZIF-4 by hot pressing.^3^ Direct sintering is generally expected to (i) have good dimensional accuracy even for complicated shapes and (ii) increase the density of the material without impairing its crystallinity. Recent studies have reported the thermal transformation behaviors of some MOFs;^35,36^ however, the details of the sintering behavior in CP sintering and its relationship with the physical properties of the CP itself have not been adequately analyzed. Consequently, the optimization of the sintering conditions and assessment of the scope of its application are difficult. In the hot-press method employed by Widmer et al.,^3^ the sintering behavior cannot be adequately understood because both the densification by pressurization and sintering by heat simultaneously occur.

Therefore, this study is aimed at elucidating the sintering behavior in the direct sintering of CPs, after compaction, from their powder form. Generally, in the sintering of metals and ceramics, the optimum sintering temperature is determined based on the melting point of the material. Furthermore, in CPs, the sintering behavior is believed to be related to the melting point and whether or not a melting point exists. Therefore, a melting CP ([Zn(HPO_4_)(H_2_PO_4_)_2_]·2H_2_Im, ZPI)^26,37^ and a non-melting CP (ZIF-8^38^) are used in this study, and the differences between these two CPs are discussed (Table 1).

**Table 1:** Comparison of the CPs/MOFs in the Present Study^a^

	ZPI	ZIF-8
composition	[Zn(HPO_4_)(H_2_PO_4_)_2_]·2H_2_Im	Zn(mIm)_2_
structural dimension	1D	3D
melting point	154 °C^26^	
decomposition temperature	200 °C^37^	400 °C^39^

a Im = imidazole and mIm = 2-methylimidazole.

## 2 Results

### 2.1 Thermogravimetric/Differential Thermal Analysis of the ZPI and ZIF-8 Powders

**Figure 1:**
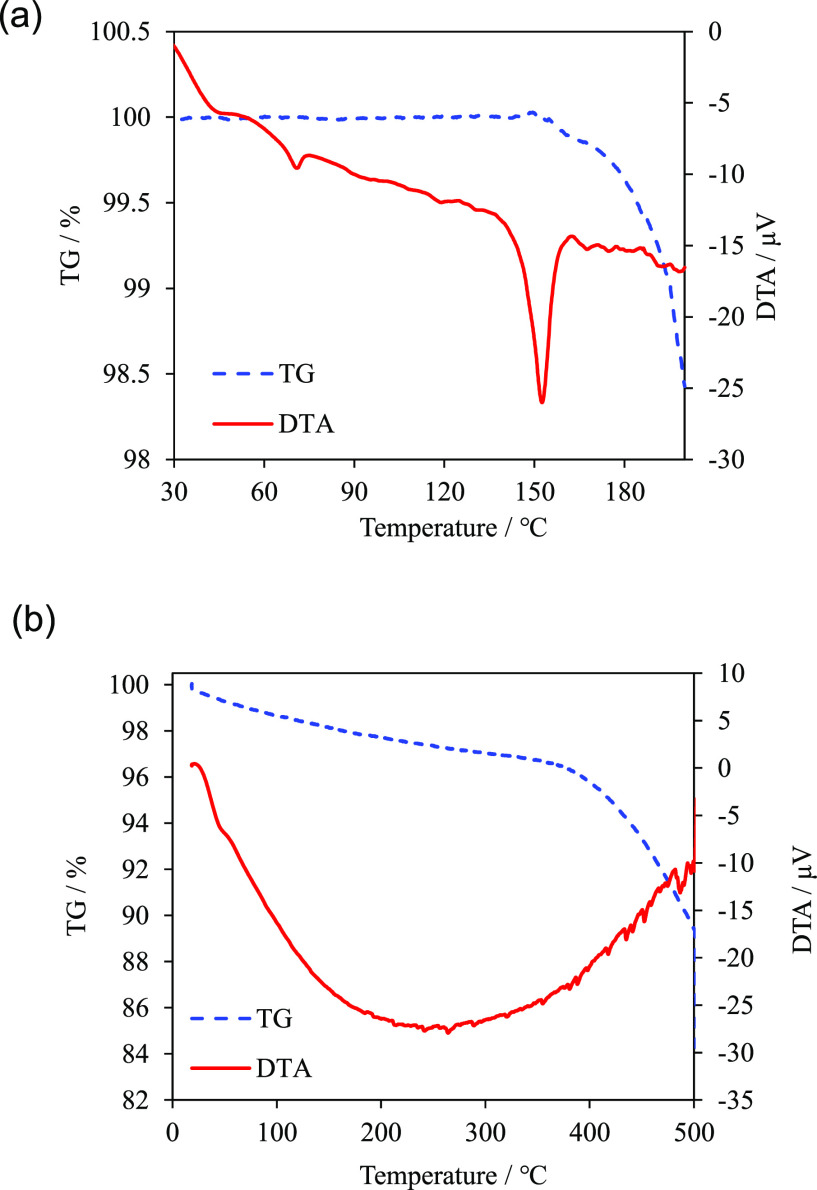
Thermogravimetric (TG) and differential thermal analysis (DTA) results for (a) ZPI and (b) ZIF-8.

Figure 1 shows the results of the TG/DTA analysis. The melting point of the synthesized ZPI is ∼150 °C, and thermal decomposition is observed to occur at ∼160 °C. These results are consistent with those of previous reports.^26^ In ZIF-8, solvent release is observed over a wide range of temperatures, starting from 27 °C (room temperature) to approximately 300 °C, while thermal decomposition is observed at approximately 400 °C. This result is in agreement with that of a previous report.^39^ Based on these results, the sintering temperatures are maintained below 110 °C for ZPI and below 270 °C for ZIF-8 to ensure that the sintering temperatures are lower than the melting point and decomposition temperature.

### 2.2 Displacement in Sintering of ZPI and ZIF-8 Green Compacts

**Figure 2:**
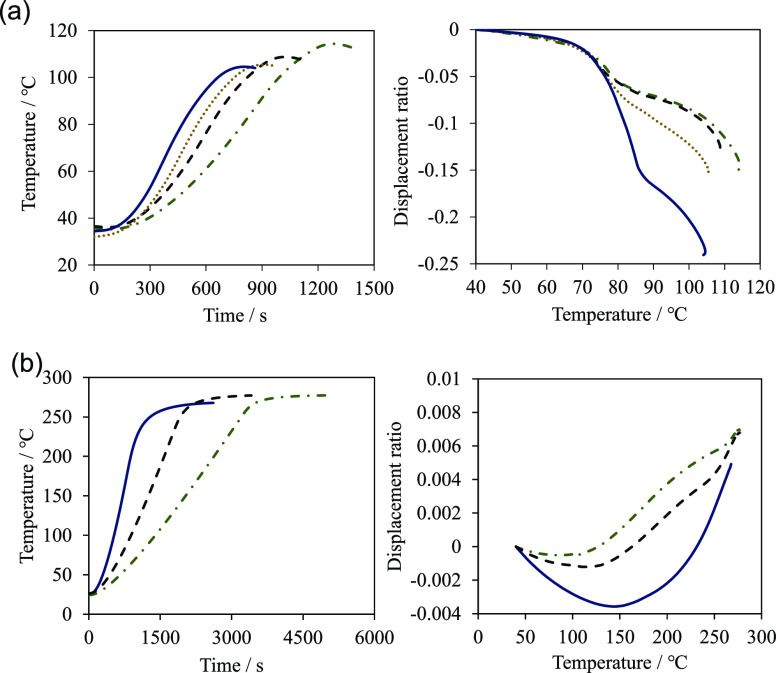
Heating rate and shrinkage behavior of (a) ZPI and (b) ZIF-8. The same line type is the corresponding sample in the left and right figure, respectively.

Figure 2 shows the heating rate and the shrinkage behavior during sintering of the green compacts. ZPI monotonically shrinks with temperature, and ultimately, a shrinkage of 10–20% is observed. Additionally, only a small difference is observed in the displacement resulting from a change in the heating rate up to approximately 80 °C. However, above 80 °C, a faster increase in temperature results in a greater observed shrinkage. Comparatively, ZIF-8 only slightly shrinks (less than 1%) at temperatures up to approximately 150 °C. Beyond approximately 150 °C, no further shrinkage is observed owing to the effects of thermal expansion. Additionally, the shrinkage behavior of ZIF-8 is dependent on the heating rate from the beginning of the process, with greater shrinkage observed for the faster heating rate.

### 2.3 Physical Characterization of the ZPI and ZIF-8 Sintered Compacts

**Figure 3:**
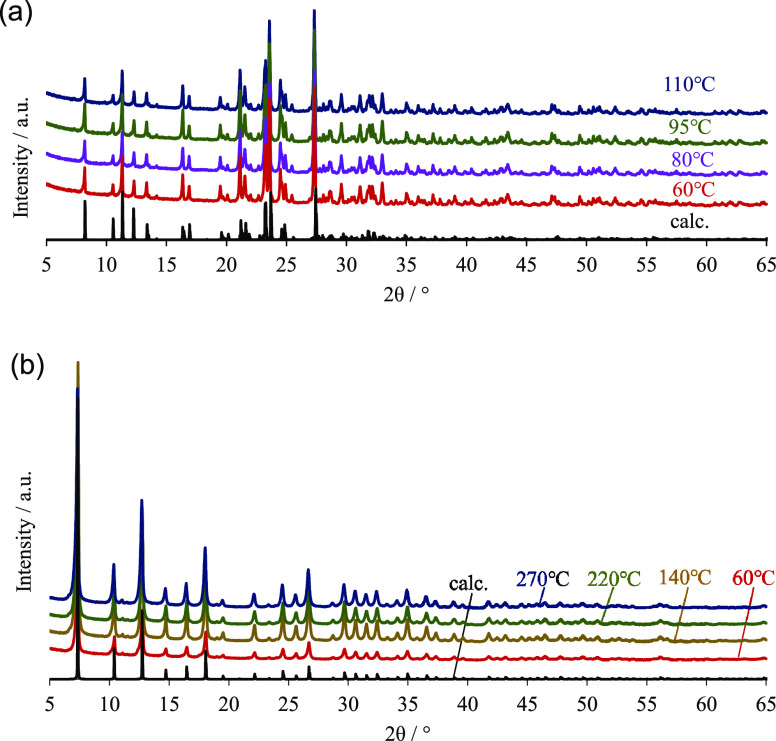
X-ray diffraction patterns of the (a) ZPI and (b) ZIF-8 bulk samples.

**Figure 4:**
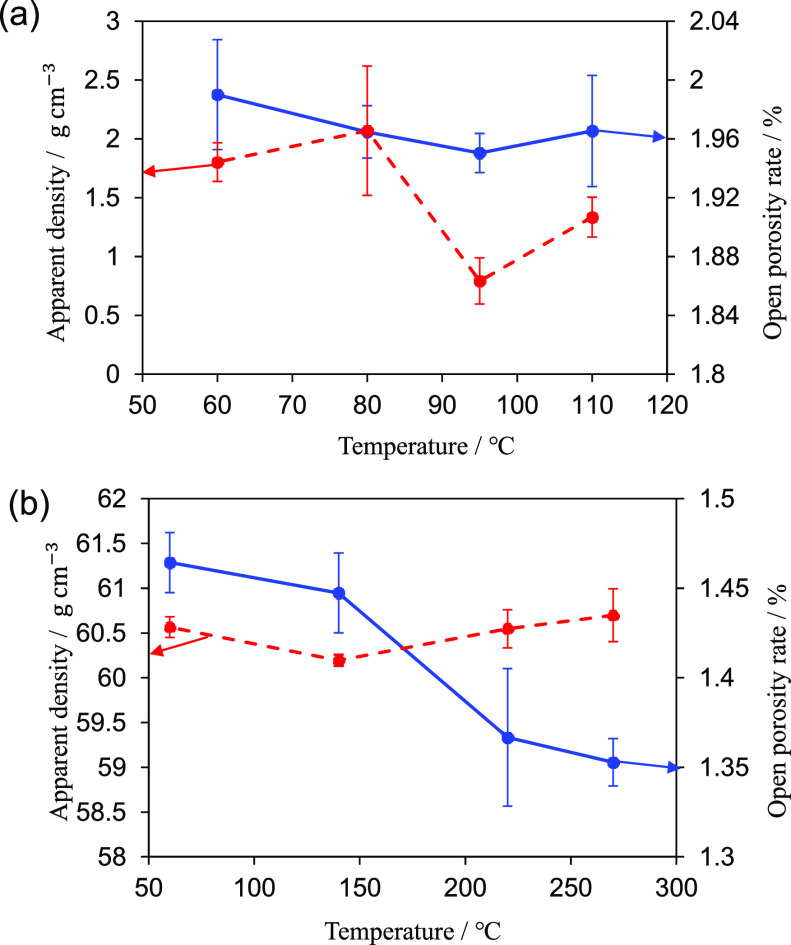
Temperature dependence of apparent density and open porosity rate for (a) ZPI and (b) ZIF-8.

**Figure 5:**
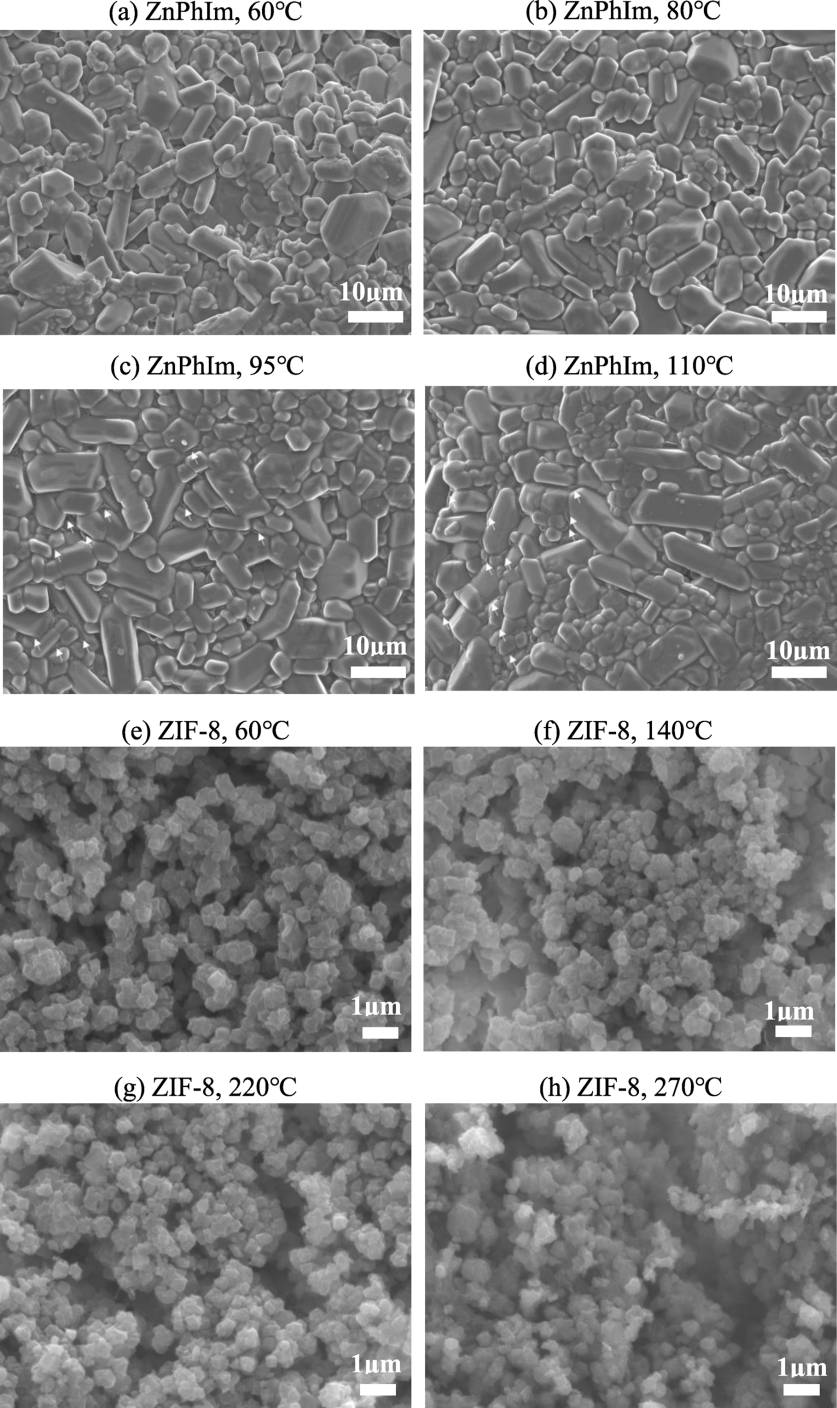
Scanning electron microscopy images of bulk samples: (a–d) ZPI and (e–h) ZIF-8. The arrows in the figure indicate where localized melting is observed at the primary particle interface.

Figure 3 shows the XRD patterns of ZPI and ZIF-8 after sintering at different temperatures. No noticeable change is observed in the XRD patterns at any temperature, and the original crystal structures are maintained. Figure 4 shows the temperature dependence of the apparent density and open porosity. In ZPI, the open porosity either changes negligibly or slightly decreases as the sintering temperature is increased. On the contrary, in ZIF-8, the open porosity significantly decreases upon increasing the sintering temperature. Furthermore, the apparent density of ZIF-8 only slightly changes as the sintering temperature is increased. Conversely, in ZPI, the apparent density does not noticeably change up to 80 °C but significantly decreases above 95 °C. Figure 5 shows the scanning electron microscopy (SEM) image of the sintered body at each sintering temperature. Almost no change is observed in ZIF-8 at any of the tested sintering temperatures. In ZPI, there is almost no change compared to the green compact state at a low temperature (below 80 °C), whereas at a high temperature (80 °C or higher), localized melting is observed between the primary particles (indicated by the arrows in Figure 5), although no significant change is observed in the primary particles themselves.

## 3 Discussion

### 3.1 Mechanism of the Sintering Process

In the thermomechanical analysis (TMA) results, only a small difference is observed in the displacement of ZPI at different heating rates up to approximately 80 °C; however, at temperatures above 80 °C, noticeable differences in shrinkage behavior are observed depending on the heating rate; faster heating rates correspond to greater shrinkage. Based on the SEM images, localized melting between the primary particles occurs in the latter temperature range. Therefore, it can be theorized that the shrinkage observed at the sintering temperatures above 80 °C is due to this localized melting. According to the initial sintering theory in ceramic sintering, the faster the heating rate, the greater the total shrinkage after sintering.^40,41^ This is because both volume and grain boundary diffusion have a greater contribution to shrinkage than surface diffusion. Additionally, the faster the heating rate during the sintering process, the more active the volume and grain boundary diffusions. These mechanisms are triggered at relatively high temperatures, while surface diffusion mainly functions at lower temperatures. Therefore, in the case of ZPI, although the sintering mechanism itself is significantly different from that of ceramics, a mechanism does exist at temperatures below 80 °C with a smaller contribution to shrinkage than with localized melting that is active above 80 °C. The details of this low temperature sintering mechanism remain to be clarified; nevertheless, based on the master sintering curve theory,^42,43^ the activation energy is estimated to be approximately 0.7 kJ/mol (Figure S4). To determine the mechanism, fundamental data such as the kinetics of mass transport or deformation in CPs are necessary. One possible explanation of this mechanism might be the atomic rearrangement of internal ZPI crystals; however, this should be clarified in future research.

In ZIF-8, the faster the heating rate from the start of the process, the greater the shrinkage at any given temperature. This tendency is the same as that of ZPI above 80 °C. Therefore, it is conceivable that even in ZIF-8, a sintering mechanism exists that significantly contributes to shrinkage only at temperatures near or higher than the initial temperature. However, as shown in Figure 5, no significant change is observed in the SEM images, and hence, a detailed mechanism for shrinkage cannot be proposed in this study. In addition, no significant differences are observed between the SEM images after TMA for the maximum and minimum heating rates in both ZPI and ZIF-8 (Figure S5).

### 3.2 Decrease in Apparent Density with the Increasing Sintering Temperature

**Figure 6:**
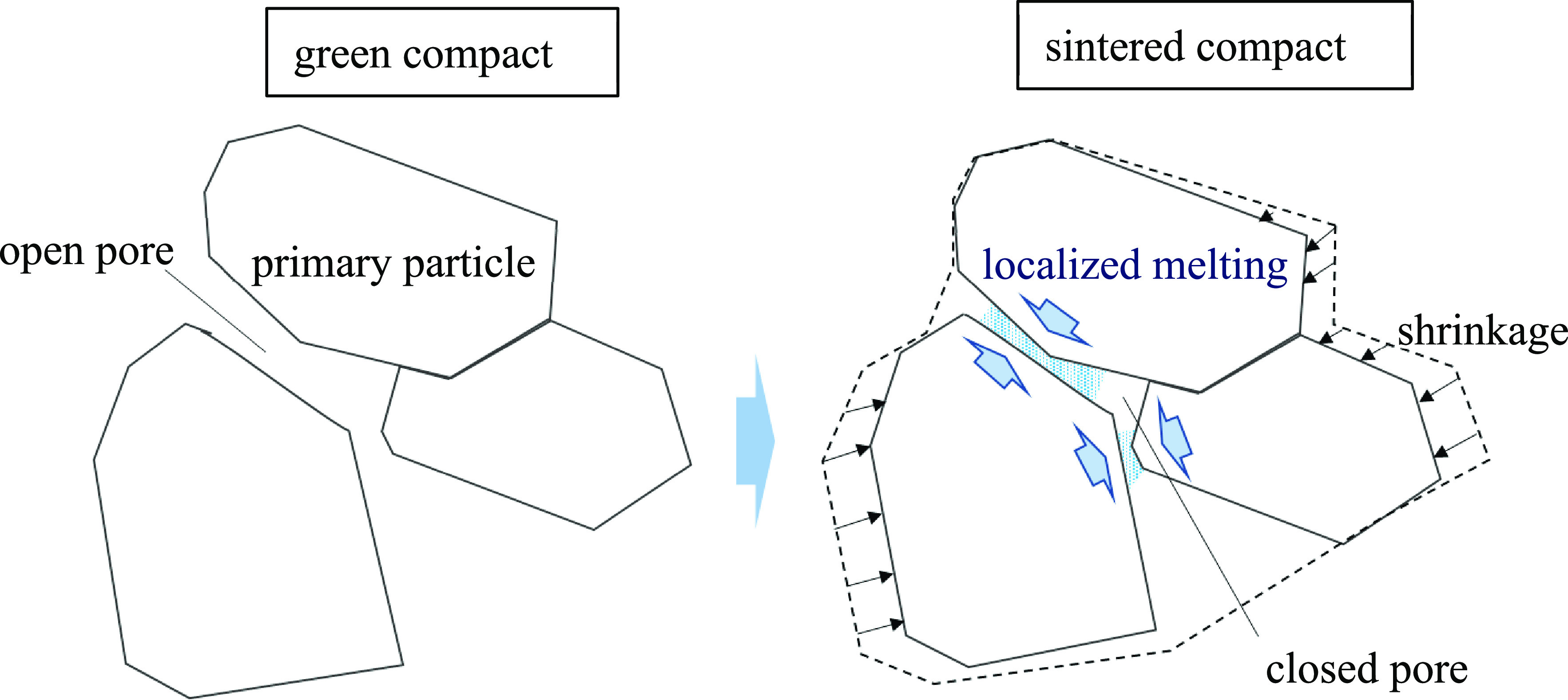
Schematic illustration of the sintering process of ZPI.

Generally, in the sintering of metals and ceramics, the apparent density increases and the open porosity decreases with an increase in sintering temperature.^44^ In ZIF-8, the observed results are consistent with this tendency (Figure 4). In contrast, in ZPI, as the sintering temperature increases, a decrease in apparent density is observed above 95 °C. Because solvent release is not observed during the sintering of ZPI, as shown in Figure 1a, the decrease in the apparent density might be due to the change in the microstructure of ZPI. One possible explanation for this is the localized melting at the interface between the primary particles in this temperature range; this introduces new closed pores, thereby increasing the apparent volume and hence decreasing the apparent density (Figure 6).

## 4 Conclusions

In this study, via simple compaction and subsequent sintering, a bulk body was obtained for both melting ZPI and non-melting ZIF-8 without the loss of macroscopic crystallinity. In particular, a rigid bulk body was obtained for ZPI. Shrinkage occurred in both samples during sintering, but the shrinkage rate of ZPI was in the range of 10–20%, whereas that of ZIF-8 was less than 1%. Additionally, the sintering mechanisms of ZPI and ZIF-8 varied between the low and high temperatures. In the case of ZPI, the localized melting between the primary particles was the dominant mechanism on the high-temperature side. However, the sizable shrinkage did not result in an increase in density. A decrease in the apparent density of ZPI was observed as the sintering temperature was increased. This indicates the introduction of new closed pores as a result of localized melting. In the case of ZIF-8, the open porosity significantly decreased with the increasing temperature, which is consistent with the usual tendency in the sintering of metals and ceramics, though the sintering mechanism is not clarified yet and is a subject for future research. We believe that the findings of this study are important from both the scientific and industrial perspectives because they deepen our understanding of CP sintering phenomena and can prove useful for process optimization in industrial applications.

## 5 Experimental Procedure

### 5.1 Materials

Zinc oxide (99.9%, 5 μm), imidazole (98%), ethanol (≥99.5%), and phosphoric acid (≥85%) were purchased from Fuji Film Wako Pure Chemical Industries, Ltd., Japan, and used in the same condition as they were received. For ZIF-8, Basolite Z1200 was purchased from Sigma-Aldrich, Japan, and activated by immersing in methanol for 24 h, followed by vacuum drying at 100 °C for 60 h.

### 5.2 Synthesis of ZPI

The synthesis of ZPI was based on a previously reported method.^4^ Zinc oxide (1620 mg, 20 mmol), imidazole (2720 mg, 40 mmol), ethanol (10 mL), and phosphoric acid (4.1 mL, 60 mmol) were placed in an agate mortar and ground for 20 min. The resultant powder was vacuum-dried at 100 °C for 47 h to obtain ZPI (Figure S1).

### 5.3 Characterization of the Powder and Sintered Body of ZPI and ZIF-8

Powder X-ray diffraction patterns were obtained using Rigaku Ultima IV with CuKα rays, a step size of 0.02°, and a scan speed of 10 °C/min. SEM images were obtained using a JEOL JSM-7000F microscope at an acceleration voltage of 10 kV. TG/DTA was performed using Rigaku D-DSC8230/TG8120IRH under nitrogen flow from 27 °C (room temperature) to 250 and 500 °C for ZPI and ZIF-8, respectively, at a heating rate of 5 °C/min. The apparent density and open porosity rate of the sintered body were measured via the Archimedes method using kerosene. The average of three measurements was recorded.

### 5.4 Powder Compacting and Sintering

ZPI and ZIF-8 powders (400 and 200 mg, respectively) were compacted with a compressive force of 10 kgf for 30 s to obtain a green compact of φ 10 mm × *t* 2 mm (Figure S2). The green compact was sintered using a tubular furnace under Ar flow to achieve the target temperature, which was maintained for 1 h at a heating rate of 20 °C/min. Based on the TGA results, the sintering temperatures were set to 60, 80, 95, and 110 °C for ZPI and 60, 140, 220, and 270 °C for ZIF-8. Figure S3 shows a photograph of the sintered body. While the ZPI bulk is rigid, the ZIF-8 bulk is slightly brittle.

### 5.5 Thermomechanical Analysis

TMA was conducted using a Seiko Instruments EXSTAR TMA/SS6100 system at a probe pressure of 10 mN and at three and four different heating rates for ZIF-8 and ZPI, respectively, under Ar flow. We define the displacement ratio as , where *l*, *l*_0_, and *l*_1_ denote the displacement, the initial sample length, and the displacement at 40 °C, respectively. The activation energy of a sintering mechanism is estimated based on the master sintering curve theory.^42,43^ We define *t*, *T*, *Q*, and *R* as the sintering time (s), absolute temperature (K), activation energy (kJ/mol), and gas constant, respectively. We then express θ(*t*, *T*(*t*)) as  and estimate an activation energy to minimize the following sum of residual squares (SRS)
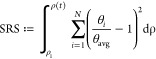
where θ_*i*_ and θ_avg_ denote the θ of the *i*-th sintering process and the average value of all sintering processes, respectively. Instead of the initial density ρ_1_ and the density at time *t* ρ(*t*), the displacement ratio at 40 °C and time *t* may be respectively used.

## Supporting Information Available

Photos of the synthesized ZPI powder, ZPI green compact, and the sintered bodies; criteria for determination of overlap of individual master sintering curves; and SEM images of bulk samples after TMA with different heating rates: (a,b) ZPI and (c,d) ZIF-8 (PDF)

## References

[ref1] Furukawa H.; Cordova K. E.; O’Keeffe M.; Yaghi O. M. The Chemistry and Applications of Metal-Organic Frameworks. Science 2013, 341, 1230444 10.1126/science.1230444. 23990564

[ref2] Küsgens P.; Zgaverdea A.; Fritz H. G.; Siegle S.; Kaskel S. Metal-Organic Frameworks in Monolithic Structures. J. Am. Ceram. Soc. 2010, 93, 2476–2479. 10.1111/j.1551-2916.2010.03824.x.

[ref3] Widmer R. N.; Lampronti G. I.; Kunz B.; Battaglia C.; Shepherd J. H.; Redfern S. A. T.; Bennett T. D. Manufacturing Macroporous Monoliths of Microporous Metal-Organic Frameworks. ACS Appl. Nano Mater. 2018, 1, 497–500. 10.1021/acsanm.7b00335.

[ref4] Shekhah O.; Wang H.; Kowarik S.; Schreiber F.; Paulus M.; Tolan M.; Sternemann C.; Evers F.; Zacher D.; Fischer R. A.; Wöll C. Step-by-Step Route for the Synthesis of Metal-Organic Frameworks. J. Am. Chem. Soc. 2007, 129, 15118–15119. 10.1021/ja076210u. 18020338

[ref5] Otsubo K.; Haraguchi T.; Sakata O.; Fujiwara A.; Kitagawa H. Step-by-Step Fabrication of a Highly Oriented Crystalline Three-Dimensional Pillared-Layer-Type Metal–Organic Framework Thin Film Confirmed by Synchrotron X-ray Diffraction. J. Am. Chem. Soc. 2012, 134, 9605–9608. 10.1021/ja304361v. 22650356

[ref6] Yao J.; Wang H. Zeolitic Imidazolate Framework Composite Membranes and Thin Films: Synthesis and Applications. Chem. Soc. Rev. 2014, 43, 4470–4493. 10.1039/C3CS60480B. 24668302

[ref7] Falcaro P.; Okada K.; Hara T.; Ikigaki K.; Tokudome Y.; Thornton A. W.; Hill A. J.; Williams T.; Doonan C.; Takahashi M. Centimetre-Scale Micropore Alignment in Oriented Polycrystalline Metal-Organic Framework Films via Heteroepitaxial Growth. Nat. Mater. 2017, 16, 342–348. 10.1038/nmat4815. 27918565

[ref8] Liu Y.; Hu E.; Khan E. A.; Lai Z. Synthesis and Characterization of ZIF-69 Membranes and Separation for CO2/CO Mixture. J. Membr. Sci. 2010, 353, 36–40. 10.1016/j.memsci.2010.02.023.

[ref9] Ji H.; Hwang S.; Kim K.; Kim C.; Jeong N. C. Direct *In Situ* Conversion of Metals into Metal-Organic Frameworks: A Strategy for the Rapid Growth of MOF Films on Metal Substrates. ACS Appl. Mater. Interfaces 2016, 8, 32414–32420. 10.1021/acsami.6b12755. 27933821

[ref10] Guo H.; Zhu G.; Hewitt I. J.; Qiu S. ‘Twin Copper Source’ Growth of Metal-Organic Framework Membrane: Cu_3_(BTC)_2_ with High Permeability and Selectivity for Recycling H_2_. J. Am. Chem. Soc. 2009, 131, 1646–1647. 10.1021/ja8074874. 19159223

[ref11] Khaletskaya K.; Turner S.; Tu M.; Wannapaiboon S.; Schneemann A.; Meyer R.; Ludwig A.; Van T.; Gustaaf F.; Roland A. Self-Directed Localization of ZIF-8 Thin Film Formation by Conversion of ZnO Nanolayers. Adv. Funct. Mater. 2014, 24, 4804–4811. 10.1002/adfm.201400559.

[ref12] Demessence A.; Boissière C.; Grosso D.; Horcajada P.; Serre C.; Férey G.; Soler-Illia G. J. A. A.; Sanchez C. Adsorption Properties in High Optical Quality nanoZIF-8 Thin Films with Tunable Thickness. J. Mater. Chem. 2010, 20, 7676–7681. 10.1039/c0jm00500b.

[ref13] Chen Z.; Wang R.; Ma T.; Wang J. L.; Duan Y.; Dai Z. Z.; Xu J.; Wang H. J.; Yuan J.; Jiang H. L.; Yin Y. W.; Li X. G.; Gao M. R.; Yu S. H. Large-Area Crystalline Zeolitic Imidazolate Framework Thin Films. Angew. Chem., Int. Ed. 2021, 60, 14124–14130. 10.1002/anie.202104366. 33856098

[ref14] Stassen I.; Styles M.; Grenci G.; Gorp H. V.; Vanderlinden W.; Feyter S. D.; Falcaro P.; Vos D. D.; Vereecken P.; Ameloot R. Chemical Vapour Deposition of Zeolitic Imidazolate Framework Thin Films. Nat. Mater. 2016, 15, 304–310. 10.1038/nmat4509. 26657328

[ref15] Qiu S.; Xue M.; Zhu G. Metal-Organic Framework Membranes: From Synthesis to Separation Application. Chem. Soc. Rev. 2014, 43, 6116–6140. 10.1039/C4CS00159A. 24967810

[ref16] Li W. Metal-Organic Framework Membranes: Production, Modification, and Applications. Prog. Mater. Sci. 2019, 100, 21–63. 10.1016/j.pmatsci.2018.09.003.

[ref17] Lin Y. S. Metal Organic Framework Membranes for Separation Applications. Curr. Opin. Chem. Eng. 2015, 8, 21–28. 10.1016/j.coche.2015.01.006.

[ref18] Kang Z.; Fan L.; Sun D. Recent Advances and Challenges of Metal-Organic Framework Membranes for Gas Separation. J. Mater. Chem. A 2017, 5, 10073–10091. 10.1039/C7TA01142C.

[ref19] Hong W. Y.; Perera S. P.; Burrows A. D. Manufacturing of Metal-Organic Framework Monoliths and Their Application in CO 2 Adsorption. Microporous Mesoporous Mater. 2015, 214, 149–155. 10.1016/j.micromeso.2015.05.014.

[ref20] Tian T.; Velazquez-Garcia J.; Bennett T. D.; Fairen-Jimenez D. Mechanically and Chemically Robust ZIF-8 Monoliths with High Volumetric Adsorption Capacity. J. Mater. Chem. A 2015, 3, 2999–3005. 10.1039/C4TA05116E.

[ref21] Ye J. W.; Zhou X.; Wang Y.; Huang R. K.; Zhou H. L.; Cheng X. N.; Ma Y.; Zhang J. P. Room-Temperature Sintered Metal-Organic Framework Nanocrystals: A New Type of Optical Ceramics. Sci. China Mater. 2018, 61, 424–428. 10.1007/s40843-017-9184-1.

[ref22] Tricarico M.; Tan J. C. Mechanical Properties and Nanostructure of Monolithic Zeolitic Imidazolate Frameworks: A Nanoindentation, Nanospectroscopy, and Finite Element Study. Mater. Today Nano 2022, 17, 100166 10.1016/j.mtnano.2021.100166.

[ref23] Bazer-Bachi D.; Assié L.; Lecocq V.; Harbuzaru B.; Falk V. Towards Industrial Use of Metal-Organic Framework: Impact of Shaping on the MOF Properties. Powder Technol. 2014, 255, 52–59. 10.1016/j.powtec.2013.09.013.

[ref24] Hou J.; Sapnik A. F.; Bennett T. D. Metal-Organic Framework Gels and Monoliths. Chem. Sci. 2020, 11, 310–323. 10.1039/C9SC04961D. 32153752 PMC7021205

[ref25] Duan C.; Yu Y.; Li J.; Li L.; Huang B.; Chen D.; Xi H. Recent Advances in the Synthesis of Monolithic Metal-Organic Frameworks. Sci. China Mater. 2021, 64, 1305–1319. 10.1007/s40843-020-1585-1.

[ref26] Umeyama D.; Horike S.; Inukai M.; Itakura T.; Kitagawa S. Reversible Solid-to-Liquid Phase Transition of Coordination Polymer Crystals. J. Am. Chem. Soc. 2015, 137, 864–870. 10.1021/ja511019u. 25530162

[ref27] Li S.; Limbach R.; Longley L.; Shirzadi A. A.; Walmsley J. C.; Johnstone D. N.; Midgley P. A.; Wondraczek L.; Bennett T. D. Mechanical Properties and Processing Techniques of Bulk Metal-Organic Framework Glasses. J. Am. Chem. Soc. 2019, 141, 1027–1034. 10.1021/jacs.8b11357. 30582804

[ref28] Hou J.; Ashling C. W.; Collins S. M.; Krajnc A.; Zhou C.; Longley L.; Johnstone D. N.; Chater P. A.; Li S.; Coulet M. V.; Llewellyn P. L.; Coudert F. X.; Keen D. A.; Midgley P. A.; Chen V.; Bennett T. D. Metal-organic framework crystal-glass composites. Nat. Commun. 2019, 10, 2580 10.1038/s41467-019-10470-z. 31189892 PMC6561910

[ref29] Ashling C. W.; Johnstone D. N.; Widmer R. N.; Hou J.; Collins S. M.; Sapnik A. F.; Bumstead A. M.; Midgley P. A.; Chater P. A.; Keen D. A.; Bennett T. D. Synthesis and Properties of a Compositional Series of MIL-53(Al) Metal–Organic Framework Crystal-Glass Composites. J. Am. Chem. Soc. 2019, 141, 15641–15648. 10.1021/jacs.9b07557. 31491080 PMC7007233

[ref30] Longley L.; Collins S. M.; Zhou C.; Smales G. J.; Norman S. E.; Brownbill N. J.; Ashling C. W.; Chater P. A.; Tovey R.; Schonlieb C. B.; Headen T. F.; Terrill N. J.; Yue Y.; Smith A. J.; Blanc F.; Keen D. A.; Midgley P. A.; Bennett T. D. Liquid phase blending of metal-organic frameworks. Nat. Commun. 2018, 9, 2135 10.1038/s41467-018-04553-6. 29907760 PMC6004012

[ref31] Tuffnell J. M.; Ashling C. W.; Hou J.; Li S.; Longley L.; Rios Gomez M. L.; Bennett T. D. Novel metal-organic framework materials: blends, liquids, glasses and crystal-glass composites. Chem. Commun. 2019, 55, 8705–8715. 10.1039/C9CC01468C. 31045184

[ref32] Horike S.; Nagarkar S. S.; Ogawa T.; Kitagawa S. A New Dimension for Coordination Polymers and Metal-Organic Frameworks: Towards Functional Glasses and Liquids. Angew. Chem., Int. Ed. 2020, 59, 6652–6664. 10.1002/anie.201911384. 31631497

[ref33] Ma N.; Horike S. Metal-Organic Network-Forming Glasses. Chem. Rev. 2022, 122, 4163–4203. 10.1021/acs.chemrev.1c00826. 35044749

[ref34] Bennett T. D.; Horike S. Liquid, Glass and Amorphous Solid States of Coordination Polymers and Metal-Organic Frameworks. Nat. Rev. Mater. 2018, 3, 431–440. 10.1038/s41578-018-0054-3.

[ref35] Widmer R. N.; Lampronti G. I.; Anzellini S.; Gaillac R.; Farsang S.; Zhou C.; Belenguer A. M.; Wilson C. W.; Palmer H.; Kleppe A. K.; Wharmby M. T.; Yu X.; Cohen S. M.; Telfer S. G.; Redfern S. A. T.; Coudert F. X.; MacLeod S. G.; Bennett T. D. Pressure promoted low-temperature melting of metal-organic frameworks. Nat. Mater. 2019, 18, 370–376. 10.1038/s41563-019-0317-4. 30886398

[ref36] Widmer R. N.; Lampronti G. I.; Chibani S.; Wilson C. W.; Anzellini S.; Farsang S.; Kleppe A. K.; Casati N. P. M.; MacLeod S. G.; Redfern S. A. T.; Coudert F. X.; Bennett T. D. Rich Polymorphism of a Metal-Organic Framework in Pressure-Temperature Space. J. Am. Chem. Soc. 2019, 141, 9330–9337. 10.1021/jacs.9b03234. 31117654 PMC7007208

[ref37] Horike S.; Umeyama D.; Inukai M.; Itakura T.; Kitagawa S. Coordination-Network-Based Ionic Plastic Crystal for Anhydrous Proton Conductivity. J. Am. Chem. Soc. 2012, 134, 7612–7615. 10.1021/ja301875x. 22512400

[ref38] Park K. S.; Ni Z.; Côté A. P.; Choi J. Y.; Huang R.; Uribe-Romo F. J.; Chae H. K.; O’Keeffe M.; Yaghi O. M. Exceptional Chemical and Thermal Stability of Zeolitic Imidazolate Frameworks. Proc. Natl. Acad. Sci. U. S. A. 2006, 103, 10186–10191. 10.1073/pnas.0602439103. 16798880 PMC1502432

[ref39] Tran U. P. N.; Le K. K. A.; Phan N. T. S. Expanding Applications of Metal-Organic Frameworks: Zeolite Imidazolate Framework ZIF-8 as an Efficient Heterogeneous Catalyst for the Knoevenagel Reaction. ACS Catal. 2011, 1, 120–127. 10.1021/cs1000625.

[ref40] Rhee S.-H.; Do Lee J.; Kim D.-Y. Effect of Heating Rate on the Exaggerated Grain Growth Behavior of β-Si3N4. Mater. Lett. 1997, 32, 115–120. 10.1016/S0167-577X(97)00018-9.

[ref41] Ikuma Y.; Nakayama M.; Harada Y.; Hiuga T. Effect of Heating Rate on the Shrinkage of Isothermal Sintering. J. Ceram. Soc. Jpn. 1991, 99, 479–482. 10.2109/jcersj.99.479.

[ref42] Pouchly V.; Maca K. Master Sintering Curve: A Practical Approach to Its Construction. Sci. Sintering 2010, 42, 25–32. 10.2298/SOS1001025P.

[ref43] Kutty T. R. G.; Khan K. B.; Hegde P. V.; Sengupta A. K.; Majumdar S.; Kamath H. S. Determination of Activation Energy of Sintering of ThO2-U3O8 Pellets Using the Master Sintering Curve Approach. Sci. Sintering 2003, 35, 125–132. 10.2298/SOS0303125K.

[ref44] Brosnan K. H.; Messing G. L.; Agrawal D. K. Microwave Sintering of Alumina at 2.45 GHz. J. Am. Ceram. Soc. 2003, 86, 1307–1312. 10.1111/j.1151-2916.2003.tb03467.x.

