# Supplementary material for: Direct Sintering Behavior of Metal Organic Frameworks/Coordination Polymers
Source: ACS Omega. 2022 Nov 18;7(51):47906–11. doi: 10.1021/acsomega.2c05732 (PMC9798516; doi:10.1021/acsomega.2c05732)
Supplement: Supplementary file 1 — ao2c05732_si_001.pdf [file ao2c05732_si_001.pdf]

Supporting Information for

Direct Sintering Behavior of Metal Organic Frameworks/Coordination Polymers

*Izuru Miyazaki, Yumi Masuoka, Akitoshi Suzumura, Shinya Moribe, Mitsutaro Umehara**

Toyota Central R&D Labs., Inc., Nagakute, Aichi, Japan.

* Corresponding author: umehara@mosk.tytlabs.co.jp

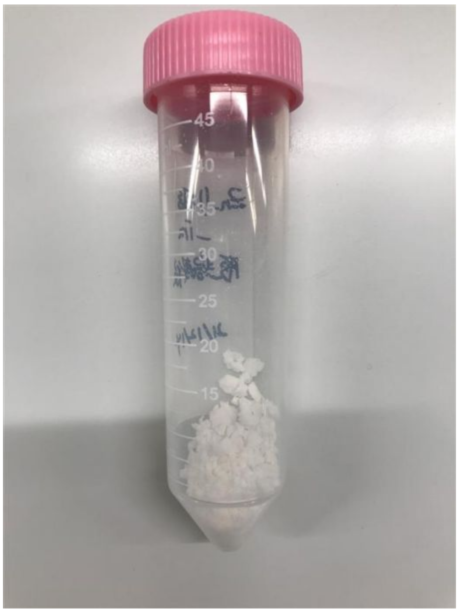

Figure. S1 The synthesized ZPI.

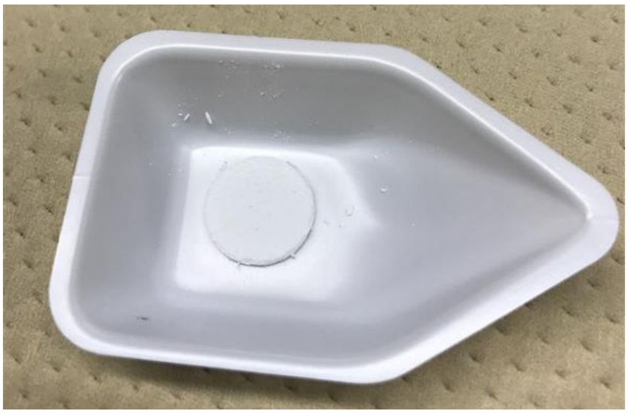

Figure. S2 ZPI green compact.

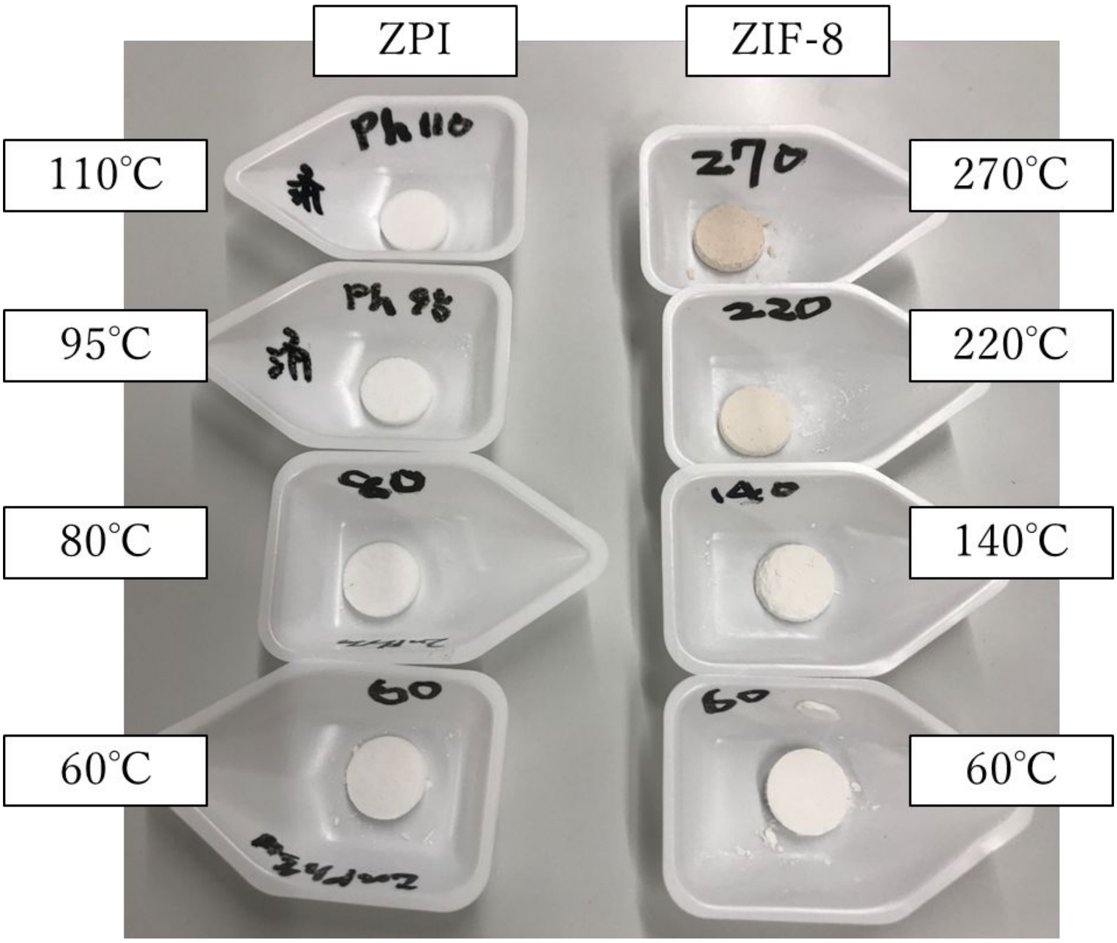

Figure. S3 The sintered bodies.

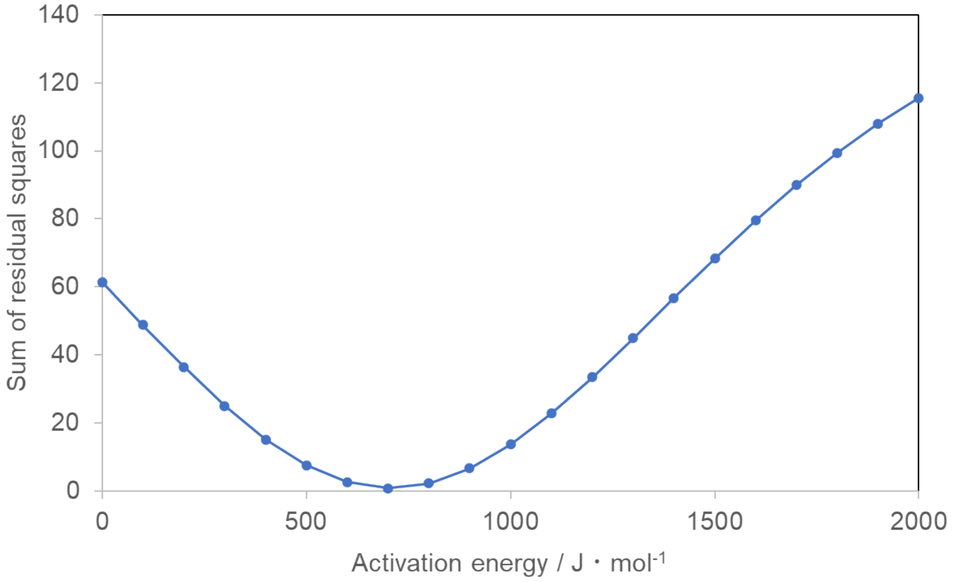

Figure. S4 Criteria for determination of overlap of individual master sintering curves.

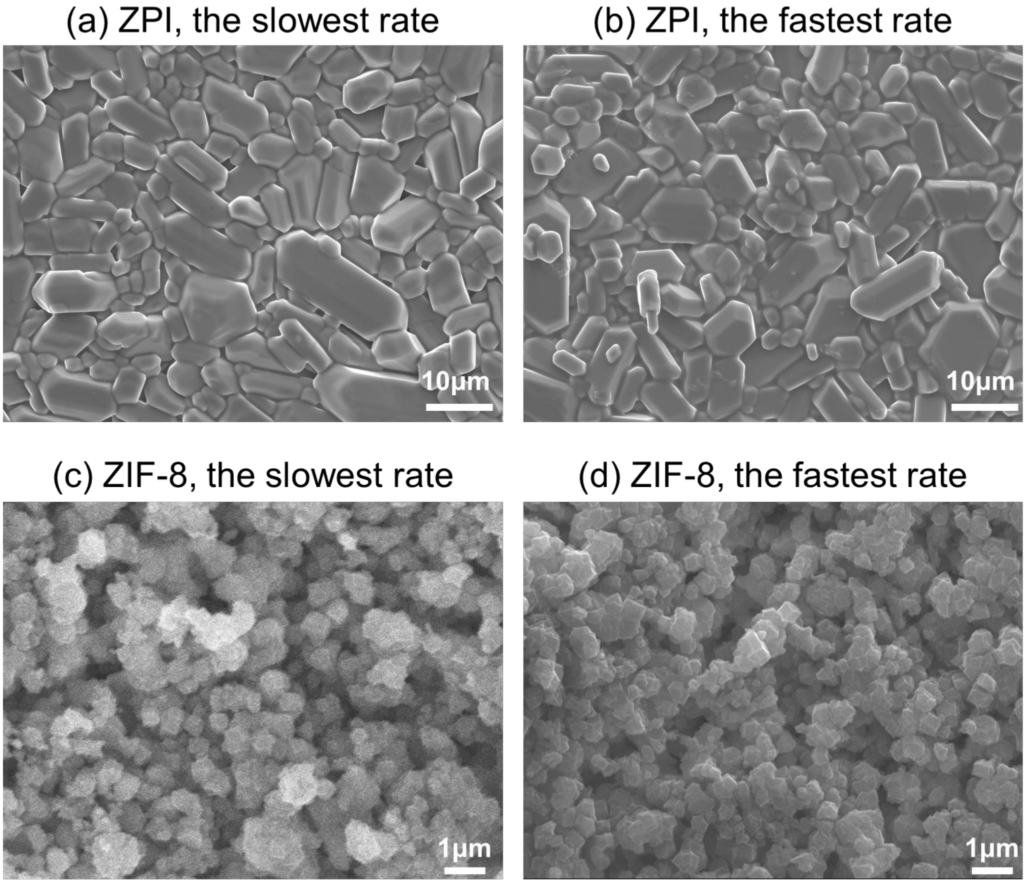

Figure. S5 SEM images of bulk samples after TMA with different heating
